# BIM在非小细胞肺癌治疗中的作用及意义

**DOI:** 10.3779/j.issn.1009-3419.2016.11.12

**Published:** 2016-11-20

**Affiliations:** 150001 哈尔滨，哈尔滨医科大学附属第二医院肿瘤内科 Department of Medical Oncology, the 2^nd^ Affiliated Hospital of Harbin Medical University, Harbin 150001, China

**Keywords:** BIM, 肺肿瘤, 酪氨酸激酶抑制剂, 化疗, BCL-2-interacting mediator of cell death (BIM), Lung neoplasms, Tyrosine kinase inhibitors, Chemotherapy

## Abstract

B细胞淋巴瘤-2促细胞凋亡（B-cell lymphoma 2 interacting mediator of cell death, *BIM*）基因作为抑癌基因，在调控细胞凋亡中起重要作用。在非小细胞肺癌（non-small cell lung cancer, NSCLC）中，BIM表达水平的下调或功能缺陷会降低酪氨酸激酶抑制剂（tyrosine kinase inhibitors, TKIs）及化疗药物的疗效并影响术后患者的预后。本文将对BIM的结构、功能以及BIM在NSCLC治疗中的作用及意义进行介绍。

## Abstract (en)

B-cell lymphoma 2 interacting mediator of cell death (BIM) plays an important role in the progress of cell apoptosis. The lowering expression level or functional defect of which may have an negative effect on the efficacy of anticancer drugs and the prognosis of postoperative patients with non-small cell lung cancer (NSCLC). This review aims to summarize the structure and function of BIM, as well as the relationship between BIM and the therapeutic efficacy of NSCLC.

肺癌是全球死亡率最高的恶性肿瘤^[1]^，其中约80%是非小细胞肺癌（non-small cell lung cancer, NSCLC）^[2]^。手术是早期NSCLC的标准治疗手段^[3]^；靶向药物的出现明显提高了晚期NSCLC的总生存^[4]^，但化疗依然在晚期NSCLC治疗中担任重要角色^[3]^。目前早期术后患者仍存在复发风险，化疗及靶向治疗面临广泛耐药，寻找耐药原因和克服耐药的方法、探索新的治疗靶点成为当前研究热点^[5]^。

BIM是B细胞淋巴瘤-2（B-cell lymphoma-2, BCL-2）家族的促凋亡成员之一，在肿瘤形成过程中起调节细胞凋亡的作用。近年来研究者发现BIM与晚期NSCLC接受表皮生长因子酪氨酸激酶抑制剂（epidermal growth factor receptor-tyrosine kinase inhibitors, EGFR-TKIs）治疗及化疗的疗效密切相关，并影响术后患者的预后。本文将简单介绍BIM的结构和功能，并阐述BIM在NSCLC治疗中的重要作用。

## 1 BIM的结构

细胞凋亡信号转导通路主要包括外源性/内源性途径，目前研究最深入的*BCL-2*基因，主要存在于线粒体外膜、核膜和内质网膜上，作为凋亡调控基因之一，在内源性凋亡途径中通过调控凋亡平衡影响细胞凋亡^[6]^。

*BIM*基因最早由O^’^Connor等^[7]^发现，是BCL-2家族促凋亡成员之一，其编码的BIM蛋白全称为B-cell lymphoma 2 interacting mediator of cell death，也称BCL2L11（BCL-2 like protein 11）。人类BIM定位于2q13，包含6个外显子，通过选择性剪接作用而形成18种不同的亚型，它们含有不同数目的碱基，也具有不同的促凋亡活性，可以表达于多种组织，比如颅脑、心脏、肾脏、肝脏、肺和卵巢等。

BIM是一种唯BH3域蛋白，这一BH3结构域是BIM与BCL-2亚族中抗凋亡成员结合的区域，在BIM的促细胞凋亡中起重要作用^[8]^。

## 2 功能

BIM广泛分布于正常组织细胞，在免疫调节、造血细胞稳态中具有重要作用^[8]^；研究提示BIM缺失可导致如白血病、自身免疫性疾病的发生；BIM在恶性肿瘤细胞内表达水平的降低会抑制肿瘤细胞凋亡，促进肿瘤的发生发展^[9-11]^。

一定的凋亡刺激信号如细胞因子撤除、化疗药物作用、紫外线照射等通过各种信号途径激活BIM分子，被激活的BIM主要通过移位于线粒体膜，然后经不同途径发挥促凋亡作用。例如，通过与抗凋亡分子相结合，间接起到促凋亡作用；或者直接活化促凋亡分子，促使其插入线粒体外膜，通过改变线粒体外膜的通透性释放细胞色素C，细胞色素C与凋亡酶激活因子（apoptotic protease activating factor-1, Apaf1）形成凋亡小体，再与凋亡蛋白caspase作用，产生caspase级联反应，导致接受刺激的细胞凋亡^[5, 12, 13]^。

BIM在肿瘤形成过程中起抑制作用，通常可被不同的上游或下游信号通路影响，表现出表达水平的上调或下调^[14]^，例如，PI3K/Akt通路、MEK/ERK1/2通路，在乳腺癌，HER2/ErbB2的过表达可以增加ERK和Akt的激活，使BIM的表达减少，阻止肿瘤细胞凋亡；在结直肠癌、肝癌和胃癌等恶性肿瘤的发生发展中也存在BIM表达水平的下调^[15]^。因此*BIM*基因的低表达或功能缺陷可以抑制肿瘤细胞凋亡，为肿瘤细胞生长提供便利条件。

## 3 BIM与NSCLC治疗

*BIM*缺失多态性指其基因外显子2和外显子3之间的内含子DNA序列有一段2, 903 bp的缺失，导致外显子3和外显子4拼接错误，使促凋亡BH3结构域的表达缺失，从而使BIM不能行使其促凋亡功能^[16]^。有研究^[17, 18]^提示BIM编码的唯BH3域蛋白在由表皮生长因子酪氨酸激酶抑制剂激发的细胞凋亡中起关键调节因子的作用。

在NSCLC中*BIM*基因表达水平的降低或BIM缺失多态性的存在会降低化疗和TKIs治疗的疗效并影响术后患者的预后，可成为治疗失败的重要原因。

### 3.1 *BIM*缺失多态性与*EGFR*突变型晚期NSCLC患者TKIs治疗疗效的关系

EGFR-TKIs的出现很大程度改善了晚期*EGFR*突变型NSCLC患者的生存期，研究^[19]^提示TKIs治疗*EGFR*突变型NSCLC的有效性和BIM的缺失多态性密切相关，并且这种缺失多态性现象只存在于亚洲人群。

Ng等^[20]^利用聚合酶链式反应（polymerase chain reaction, PCR）证实了在EGFR-TKIs耐药的*EGFR*突变型非小细胞肺癌HCC2279细胞系中存在*BIM*基因的缺失多态性，然后对有或无BIM缺失多态性患者的中位疾病无进展生存期（progression-free survival, PFS）进行了分析比较，结果包含*BIM*缺失多态性的患者表现出更短的中位PFS（分别为6.6个月和11.9个月，*P*=0.002, 7），并且多变量分析表明*BIM*缺失多态性可作为更短PFS的独立影响因素（HR=2.08, 95%CI: 1.29-3.38, *P*=0.002, 8）。

随着NSCLC靶向治疗耐药现象的不断增加，更多研究开始探索其具体耐药机制。为了评价在临床工作中检测*EGFR*突变型NSCLC患者*BIM*缺失多态性的实用价值，Isobe等^[21]^入组了日本的70例2008年-2013年接受TKIs治疗的晚期*EGFR*突变型NSCLC患者，其中18.6%（13/70）患者的外周血或组织切片检测到*BIM*缺失多态性，回顾性分析提示*BIM*缺失多态性患者表现更短的PFS（222天*vs* 533天，*P* < 0.001），因此建议检测*BIM*的基因多态性，并针对这些患者制定新的治疗措施。

Zhao等^[22]^回顾性分析中国NSCLC患者BIM状态与EGFR-TKIs治疗疗效的关系，在接受EGFR-TKIs治疗的239例患者中*BIM*缺失多态性者28例，结果含有*BIM*缺失多态性患者的中位PFS为4.7个月，无*BIM*缺失多态性者11个月（*P*=0.003）；客观反应率（objective response rate, ORR）为25% *vs* 66%（*P*=0.001），提示对于存在BIM缺失多态性的*EGFR*突变型NSCLC患者，TKIs治疗的疗效会比较差。这也证实了BIM在EGFR-TKIs激发的肿瘤细胞凋亡中的重要作用。研究还对*EGFR*突变类型（主要为19外显子缺失、L858R突变两种类型）是否会影响*BIM*缺失多态性引起短PFS进行了统计分析，结果显示外显子19缺失者mPFS为7.3个月，L858R的mPFS为4.1个月（*P*=0.377），虽然结果提示*EGFR*突变类型与*BIM*缺失多态性引起的TKIs耐药不相关，但两组治疗效果仍然较差。而这项研究入组患者中具有*BIM*缺失多态性者均为杂合子型，未发现纯合子型，这点可能对该研究造成一定限制。

Huang等^[23]^进行了一项共包含6项前瞻性或回顾性研究的*meta*分析，773例EGFR-TKIs治疗后进展的*EGFR*突变型NSCLC患者入组，并参照BIM多态性进行分层分析了*BIM*缺失多态性与EGFR-TKIs治疗效果的关系，结果显示无*BIM*缺失多态性的患者表现出更长的PFS（*P*=0.001）。

另有研究提示*BIM*缺失多态性与患者对EGFR-TKIs治疗敏感性的降低之间并非绝对相关。在Li^[24]^的研究中，接受EGFR-TKIs治疗患者的PFS与其*BIM*缺失多态性无关（缺失多态性者11.9个月*vs* 野生型10.9个月，*P*=0.160）。Xia等^[25]^及Ebi等^[26]^在各自的关于NSCLC风险与*BIM*缺失多态性之间关系的研究中也得出相似观点。这可能是由于肺癌的发生并不完全独立归因于BIM通路^[27]^。

### 3.2 BIM与晚期NSCLC患者化疗疗效的关系

含铂化疗方案在NSCLC治疗中占重要位置。研究显示在铂类耐药的肿瘤细胞内BIM呈低表达状态，在铂类敏感细胞内相反，这提示BIM表达水平与铂类化疗敏感性及耐药性相关^[28]^；另有文献^[29]^报道BIM过表达的细胞对微管毒性药物紫杉醇更敏感。Costa等^[30]^进行了一项随机的Ⅲ期临床研究，通过PCR检测BIM表达水平并探讨其与晚期NSCLC患者化疗疗效的关系，结果提示BIM高表达水平者获得更长的PFS，化疗总反应率为14.3% *vs* 11.1%（*P* < 0.000, 1）。

*BIM*缺失多态性的存在会导致其表达水平的降低，影响晚期NSCLC患者的化疗疗效。回顾性研究^[31]^显示BIM缺失多态性患者化疗后PFS相对更短（3.5个月*vs* 5.6个月，*P*=0.050），并且多因素分析提示BIM缺失多态性可作为化疗的重要预后因子（HR=2.4, *P*=0.016）。日本的一项回顾性研究分析了不同BIM多态性状态的术后复发NSCLC患者接受化疗的复发生存期（post-recurrence survival, PRS），结果*BIM*缺失多态性患者化疗的PRS明显较短（6.2个月*vs* 18.5个月，*P*=0.046），以上研究提示BIM表达水平的降低或功能缺陷与NSCLC化疗疗效密切相关^[32]^。

Xia等^[25]^还对铂类为基础的化疗所致的不良反应进行回顾性分析，结果*BIM*缺失多态性与化疗所致血小板减少症明显相关（*P*=0.048, 95%CI: 0.34-0.99）。

### 3.3 *BIM*缺失多态性对NSCLC患者术后预后的影响

既往关于*BIM*缺失多态性与NSCLC治疗的相关研究都是关于不能手术的晚期NSCLC，但是针对手术完全切除的NSCLC患者的相关研究很少。

Ebi等^[26]^分析了139例Ⅰ期接受手术切除治疗的肺癌患者的总生存期（overall survival, OS），并参考*BIM*多态性状态进行分组，结果提示无论BIM状态如何，两组患者的OS并无统计学差异（*P*=0.542, 3）。

近期Atsumi等^[32]^对日本NSCLC术后患者进行了一项回顾性分析，5年OS、无复发生存期（recurrence-free survival, RFS）及PRS为主要研究终点，结果显示与BIM野生型患者相比，*BIM*缺失多态性患者的各项主要研究终点均较短（OS：58.8% *vs* 78.9%，*P* < 0.001；RFS：9.8个月 *vs* 13.9个月，*P*=0.003；PRS：11.4个月*vs* 26.9个月，*P* < 0.001），该研究提示*BIM*缺失多态性与术后NSCLC患者复发时间正相关。并且对于分别接受EGFR-TKIs治疗、化疗以及放疗的患者，*BIM*缺失多态性者PRS相对更短。这两项研究结果不一的原因可能是检测手段的差异，另外BIM对细胞凋亡以及TKIs耐药的影响存在剂量依赖性^[28]^。

## 4 展望

*BIM*作为抑癌基因在调控细胞凋亡中起重要作用，其表达水平的降低及功能缺陷与抗肿瘤药物的耐药及不良反应相关，在应用抗肿瘤药物的同时检测BIM状态能更好地指导临床治疗。在小细胞肺癌（small cell lung cancer, SCLC）的治疗中，有许多BH3模拟物正处于临床试验阶段^[33]^，如ABT-737、ABT-263。也有研究显示PI3K抑制剂联合BH3模拟物能延长耐药SCLC患者的生存期^[34]^。

已有研究^[35]^证明BH3模拟物联合二代TKIs在增强治疗效果的同时可减少耐药性和不良反应，这提示BH3模拟物联合TKIs有望成为一种新型的治疗方法。寻找检测*BIM*缺失多态性更敏感的方法，并通过不同分子水平检测共同分析以提高精确性可作为进一步探究的方向，将为更好指导临床医生给患者选择更合适的个体化治疗方案提供更多更准确的依据。因此在未来的NSCLC治疗中，可以采用针对不同途径多个靶点的联合治疗，从而在发挥最大药物疗效的同时减少药物不良反应及耐药的发生。
